# Filipino help-seeking for mental health problems and associated barriers and facilitators: a systematic review

**DOI:** 10.1007/s00127-020-01937-2

**Published:** 2020-08-20

**Authors:** Andrea B. Martinez, Melissa Co, Jennifer Lau, June S. L. Brown

**Affiliations:** 1 Department of Behavioral Sciences, College of Arts and Sciences, University of the Philippines Manila, Manila, Philippines; 2 Department of Psychology, Institute of Psychiatry, Psychology and Neuroscience, King’s College London, London, England; 3 Health Service and Population Research Department, Institute of Psychiatry, Psychology and Neuroscience, King’s College London, London, England

**Keywords:** Help-seeking, Mental health service use, Barriers and facilitators, Mental health, Filipinos, Philippines

## Abstract

**Purpose:**

This systematic review aims to synthesise the evidence on behavioural and attitudinal patterns as well as barriers and enablers in Filipino formal help-seeking.

**Methods:**

Using PRISMA framework, 15 studies conducted in 7 countries on Filipino help-seeking were appraised through narrative synthesis.

**Results:**

Filipinos across the world have general reluctance and unfavourable attitude towards formal help-seeking despite high rates of psychological distress. They prefer seeking help from close family and friends. Barriers cited by Filipinos living in the Philippines include financial constraints and inaccessibility of services, whereas overseas Filipinos were hampered by immigration status, lack of health insurance, language difficulty, experience of discrimination and lack of acculturation to host culture. Both groups were hindered by self and social stigma attached to mental disorder, and by concern for loss of face, sense of shame, and adherence to Asian values of conformity to norms where mental illness is considered unacceptable. Filipinos are also prevented from seeking help by their sense of resilience and self-reliance, but this is explored only in qualitative studies. They utilize special mental health care only as the last resort or when problems become severe. Other prominent facilitators include perception of distress, influence of social support, financial capacity and previous positive experience in formal help.

**Conclusion:**

We confirmed the low utilization of mental health services among Filipinos regardless of their locations, with mental health stigma as primary barrier, while resilience and self-reliance as coping strategies were cited in qualitative studies. Social support and problem severity were cited as prominent facilitators.

## Introduction

Mental illness is the third most common disability in the Philippines. Around 6 million Filipinos are estimated to live with depression and/or anxiety, making the Philippines the country with the third highest rate of mental health problems in the Western Pacific Region [1]. Suicide rates are pegged at 3.2 per 100,000 population with numbers possibly higher due to underreporting or misclassification of suicide cases as ‘undetermined deaths’ [2]. Despite these figures, government spending on mental health is at 0.22% of total health expenditures with a lack of health professionals working in the mental health sector [1, 3]. Elevated mental health problems also characterise ‘overseas Filipinos’, that is, Filipinos living abroad [4]. Indeed, 12% of Filipinos living in the US suffer from psychological distress [5], higher than the US prevalence rate of depression and anxiety [1]. Long periods of separation from their families and a different cultural background may make them more prone to acculturative stress, depression, anxiety, substance use and trauma especially those who are exposed to abuse, violence and discrimination whilst abroad [6].

One crucial barrier to achieving well-being and improved mental health among both ‘local’ and overseas Filipinos is their propensity to not seek psychological help [7, 8]. Not only are help-seeking rates much lower than rates found in general US populations [9], they are also low compared to other minority Asian groups [10]. Yet, few studies have been published on Filipino psychological help-seeking either in the Philippines or among those overseas [11]. Most available studies have focused on such factors as stigma tolerance, loss of face and acculturation factors [12, 13].

To date, no systematic review of studies on Filipino psychological help-seeking, both living in the Philippines and overseas, has been conducted. In 2014, Tuliao conducted a narrative review of the literature on Filipino mental health help-seeking in the US which provided a comprehensive treatise on cultural context of Filipino help-seeking behavior [11]. However, new studies have been published since which examine help-seeking in other country contexts, such as Norway, Iceland, Israel and Canada [6, 14–16]. Alongside recent studies on local Filipinos, these new studies can provide basis for comparison of the local and overseas Filipinos [7, 8, 12, 17].

This systematic review aims to critically appraise the evidence on behavioural and attitudinal patterns of psychological help-seeking among Filipinos in the Philippines and abroad and examine barriers and enablers of their help-seeking. While the majority of studies undertaken have been among Filipino migrants especially in the US where they needed to handle additional immigration challenges, studying help-seeking attitudes and behaviours of local Filipinos is important as this may inform those living abroad [10, 13, 18]. This review aims to: (1) examine the commonly reported help-seeking attitudes and behaviors among local and overseas Filipinos with mental health problems; and (2) expound on the most commonly reported barriers and facilitators that influence their help-seeking.

## Methods

The review aims to synthesize available data on formal help-seeking behavior and attitudes of local and overseas Filipinos for their mental health problems, as well as commonly reported barriers and facilitators. Formal psychological help-seeking behavior is defined as seeking services and treatment, such as psychotherapy, counseling, information and advice, from trained and recognized mental health care providers [19]. Attitudes on psychological help-seeking refer to the evaluative beliefs in seeking help from these professional sources [20].

### Eligibility criteria

Inclusion criteria for the studies were the following: (1) those that address either formal help-seeking behavior OR attitude related to a mental health AND those that discuss barriers OR facilitators of psychological help-seeking; (2) those that involve Filipino participants, or of Filipino descent; in studies that involve multi-cultural or multi-ethnic groups, they must have at least 20% Filipino participants with disaggregated data on Filipino psychological help-seeking; (3) those that employed any type of study designs, whether quantitative, qualitative or mixed-methods; (4) must be full-text peer-reviewed articles published in scholarly journals or book chapters, with no publication date restrictions; (5) written either in English or Filipino; and (6) available in printed or downloadable format. Multiple articles based on the same research are treated as one study/paper.

Exclusion criteria were: (1) studies in which the reported problems that prompted help-seeking are medical (e.g. cancer), career or vocational (e.g., career choice), academic (e.g., school difficulties) or developmental disorders (e.g., autism), unless specified that there is an associated mental health concern (e.g., anxiety, depression, trauma); (2) studies that discuss general health-seeking behaviors; (3) studies that are not from the perspective of mental health service users (e.g., counselor’s perspective); (4) systematic reviews, meta-analyses and other forms of literature review; and (5) unpublished studies including dissertations and theses, clinical reports, theory or methods papers, commentaries or editorials.

### Search strategy and study selection

The search for relevant studies was conducted through electronic database searching, hand-searching and web-based searching. Ten bibliographic databases were searched in August to September 2018: PsychInfo, Global Health, MedLine, Embase, EBSCO**,** ProQuest**,** PubMed**,** Science Direct, Scopus and Emerald Insight. The following search terms were used: “help-seeking behavior” OR “utilization of mental health services” OR “access to mental health services” OR “psychological help-seeking” AND “barriers to help-seeking” OR “facilitators of help-seeking” AND “mental health” OR “mental health problem” OR “mental disorder” OR “mental illness” OR “psychological distress” OR “emotional problem” AND “Filipino” OR “Philippines”. Filters were used to select only publications from peer-reviewed journals. Internet searches through Google Scholar and websites of Philippine-based publications were also performed using the search term “Filipino mental health help-seeking” as well as hand-searching of reference lists of relevant studies. A total of 3038 records were obtained. Duplicates were removed and a total of 2659 records were screened for their relevance based on their titles and abstracts.

**Fig. 1:**
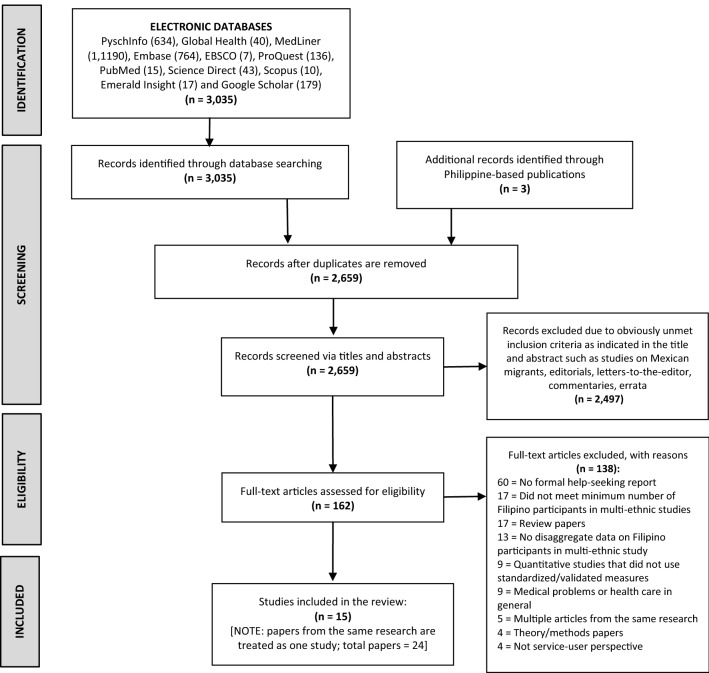
PRISMA flow diagram

Preliminary screening of titles and abstracts of articles resulted in 162 potentially relevant studies, their full-text papers were obtained and were reviewed for eligibility by two reviewers (AM and MC). Divergent opinions on the results of eligibility screening were deliberated and any further disagreement was resolved by the third reviewer (JB). A total of 15 relevant studies (from 24 papers) published in English were included in the review and assessed for quality. There were seven studies with multiple publications (two of them have 3 papers) and a core paper was chosen on the basis of having more comprehensive key study data on formal help-seeking. Results of the literature search are reported in Fig. 1 using the PRISMA diagram [21]. A protocol for this review was registered at PROSPERO Registry of the Centre for Reviews and Dissemination of the University of York (https://www.crd.york.ac.uk/PROSPERO; ID: CRD42018102836).

### Data extraction and quality assessment

Data extracted by the main author were crosschecked by a second reviewer (JB). A data extraction table with thematic headings was prepared and pilot tested for two quantitative and two qualitative studies to check data comparability. Extraction was performed using the following descriptive data: (1) study information (e.g. name of authors, publication date, study location, setting, study design, measurement tools used); (2) socio-demographic characteristics of participants (e.g. sample size, age, gender); and (3) overarching themes on psychological help-seeking behavior and attitudes, as well as barriers and facilitators of help-seeking.

Two reviewers (AM and MC) did quality assessment of the studies separately, using the following criteria: (1) relevance to the research question; (2) transparency of the methods; (3) robustness of the evidence presented; and (4) soundness of the data interpretation and analysis. Design-specific quality assessment tools were used in the evaluation of risk of bias of the studies, namely: (1) Critical Appraisal Skills Programme Qualitative Checklist [22]; and (2) Quality Assessment Tool for Quantitative Studies by the Effective Public Health Practice Project [23]. The appraisals for mixed-methods studies were done separately for quantitative and qualitative components to ensure trustworthiness [24] of the quality of each assessment.

For studies reported in multiple publications, quality assessment was done only on the core papers [25]. All the papers (*n* = 6) assessed for their qualitative study design (including the 4 mixed-methods studies) met the minimum quality assessment criteria of fair (*n* = 1) and good (*n* = 5) and were, thus, included in the review. Only 11 out of the 13 quantitative studies (including the 4 mixed-methods studies) satisfied the minimum ratings for the review, with five getting strong quality rating. The two mixed-methods studies that did not meet the minimum quality rating for quantitative designs were excluded as sources of quantitative data but were used in the qualitative data analysis because they satisfied the minimum quality rating for qualitative designs.

### Strategy for data analysis

Due to the substantial heterogeneity of the studies in terms of participant characteristics, study design, measurement tools used and reporting methods of the key findings, narrative synthesis approach was used in data analysis to interpret and integrate the quantitative and qualitative evidence [26, 27]. However, one crucial methodological limitation of studies in this review is the lack of agreement on what constitutes formal help-seeking. Some researchers include the utilization of traditional or indigenous healers as formal help-seeking, while others limit the concept to professional health care providers. As such, consistent with Rickwood and Thomas’ definition of formal help-seeking [19], data extraction and analysis were done only on those that reported utilization of professional health care providers.

Using a textual approach, text data were coded using both predetermined and emerging codes [28]. They were then tabulated, analyzed, categorized into themes and integrated into a narrative synthesis [29]. Exemplar quotations and author interpretations were also used to support the narrative synthesis. The following were the themes on barriers and facilitators of formal help-seeking: (1) psychosocial barriers/facilitators, which include social support from family and friends, perceived severity of mental illness, awareness of mental health issues, self-stigmatizing beliefs, treatment fears and other individual concerns; (2) socio-cultural barriers/facilitators, which include the perceived social norms and beliefs on mental health, social stigma, influence of religious beliefs, and language and acculturation factors; and (3) systemic/structural and economic barriers/facilitators, which include financial or employment status, the health care system and its accessibility, availability and affordability, and ethnicity, nativity or immigration status.

## Results

### Study and participant characteristics

The 15 studies were published between 2002 and 2018. Five studies were conducted in the US, four in the Philippines and one study each was done in Australia, Canada, Iceland, Israel and Norway. One study included participants working in different countries, the majority were in the Middle East. Data extracted from the four studies done in the Philippines were used to report on the help-seeking behaviors and attitudes, and barriers/facilitators to help-seeking of local Filipinos, while the ten studies conducted in different countries were used to report on help-seeking of overseas Filipinos. Nine studies were quantitative and used a cross-sectional design except for one cohort study; the majority of them used research-validated questionnaires. Four studies used mixed methods with surveys and open-ended questionnaires, and another two were purely qualitative studies that used interviews and focus group discussions. Only three studies recruited participants through random sampling and the rest used purposive sampling methods. All quantitative studies used questionnaires in measures of formal help-seeking behaviors, and western-standardized measures to assess participants’ attitudes towards help-seeking. Qualitative studies utilized semi-structured interview guides that were developed to explore the psychological help-seeking of participants.

A total of 5096 Filipinos aged 17–70 years participated in the studies. Additionally, 13 studies reported on the mean age of participants, with the computed overall mean age at 39.52 (SD 11.34). The sample sizes in the quantitative studies ranged from 70 to 2285, while qualitative studies ranged from 10 to 25 participants. Of the participants, 59% (*n* = 3012) were female which is probably explained by five studies focusing on Filipino women. Ten studies were conducted in community settings, five in health or social centre-based settings and 1 in a university (Table 1).

**Table 1:** Study and participant characteristics

Study	Participant characteristics	Location	Setting	Methodology	Questionnaire used on help-seeking	Quality assessment score
Abe-Kim et al. (2007) [10] Supplementary paper: Nguyen and Lee (2010)	508 Filipino Americans in multi-ethnic study, with 53.74% (*n* = 273) females and 46.26% (*n* = 235) males and mean age of 41.904 (SD 16.11)	US	Community-based	Quantitative Cross-sectional study design (derived from National Latino and Asian American Study, 2002–2003)	Study-specific questionnaire on help-seeking sources, need for services, treatment satisfaction	Strong*
Bernardo and Estrellado (2017) [8]	70 Filipino women, with mean age of 39.13 (SD 9.175)	Philippines	Center-based women shelters	Quantitative Cross-sectional study design	Help-seeking intention scale Locus of hope scale	Moderate*
Cabbigat and Kangas (2017)	117 Filipinos with 80.34% (*n* = 94) females and 19.66% (*n* = 23) males and mean age of 42.66 (SD 8.268)	Philippines	Local government, social welfare agencies and non-government organizations	Quantitative Cohort analytic study design	Study-specific questionnaire on help-seeking behavior Help-seeking preferences Attitudes Towards Services for Children and Adolescents (parent-report section of the child and adolescent services assessment)	Moderate*
David (2010) [34]	118 Filipino Americans with 47.46% (*n* = 56) females and 52.54% (*n* = 62) males and mean age of 30.20 (SD = 10.65)	US	Community-based	Quantitative Cross-sectional study design	Inventory of attitudes toward seeking mental health services Cultural mistrust inventory Loss of Face Questionnaire Asian value scale	Moderate*
Gong et al. (2003) [13] Supplementary papers: Abe-Kim et al., (2004) [73], Nicdao et al. (2015) [5]	2285 Filipino Americans, with 50.60% (*n* = 1156) females and 49.40% (*n* = 1128) males and mean age of 41.662 (SD 13.398)	US	Community-based	Quantitative Cross-sectional study design (derived from Filipino American Epidemiological Study, 1995–1999)	Study-specific questionnaire on help-seeking based on Kleinman (1978) typology of help-seeking sources: lay system, professional care system and folk system Research-designed questionnaire on loss of face	Moderate*
Green and Ayalon (2016) [6]	85 Filipino migrant home care workers with 86% (*n* = 73) females and 14% (*n* = 12) males and mean age of 37.04 (SD 6.70)	Israel	Community-based	Quantitative Cross-sectional study design	Study-specific questionnaire on social support, formal and informal report of abuse	Strong*
Hechanova et al. (2013) [31] Supplementary paper: Hechanova et al. (2011)	365 overseas Filipino workers with 52.88% (*n* = 193) females and 47.12% (*n* = 172) males and mean age of 33.14 (SD 7.72)	Philippines with overseas Filipino participants mostly in Middle East	Employment agencies and university-based counseling centers	Mixed methods using surveys, interviews and chat conversations	Semi-structured open-ended questionnaire Intention to seek counseling inventory (adapted version)	Weak*/ Fair**
Hermannsdottir and Aegisdottir (2016)	209 Filipino immigrants with 67% (*n* = 140) females and 33% (*n* = 69) males and mean age of 38.72 (SD 11.33)	Iceland	Community-based	Quantitative Cross-sectional study design	Psychological help-seeking attitudes and intentions Beliefs about psychological services scale Study-specific questionnaire on system barriers	Moderate*
Ho et al. (2018) [7]	175 Filipino participants for survey and focus group discussion with 45.71% (*n* = 80) females and 54.29% (*n* = 95) males and mean age of 30.49 (SD 9.70)	Multi-country study in Fiji, Cambodia and the Philippines	Community-based	Mixed methods using surveys and focus group discussions	Attitudes and understanding towards mental disorder Attitudes Toward Seeking Professional Psychological Help—Short Form and semi-structured guide for interview and focus group discussion Semi-structured interview guide for focus group discussion	Moderate */Good**
Nguyen (2011) [30] Supplementary paper: Nguyen (2012)	269 Filipino Americans in multi-ethnic study, with 57.25% (*n* = 154) females and 42.75% (*n* = 115) males and mean age of 61.8 (SD 9.9)	US	Community-based	Quantitative Cross-sectional study design (derived from California Health Interview Survey, 2001)	Study-specific questionnaire	Strong*
Shoultz et al. (2010) [32]	10 Filipino American women with age range from 34 to 52 years old	US	Women’s support agency	Mixed methods using surveys, individual interviews and focus group discussions	Semi-structured interview guide on help-seeking behavior Perceptions of the Acceptability of violence	Weak* / Good**
Straiton et al. (2018) [14] Supplementary paper: Straiton et al. (2017) [67]	14 Filipino women with mean age of 33.7	Norway	Community-based	Qualitative using in-depth interviews	Semi-structured interview guide with open-ended questions	Good**
Thompson et al. (2002a) [33] Supplement papers: Kelaher et al. (2000), Thompson et al. (2002b) [72]	487 Filipino women participated with mean age of 41.034 (SD 11.19)	Australia	Community-based	Mixed methods using multiple follow-up surveys, in-depth interviews and focus group discussions (derived from Filipina cohort of Australian Longitudinal Study on Women’s Health, 1996)	Semi-structured interview guide General Health Questionnaire	Strong*/Good**
Tuliao et al. (2016) [12] Supplementary paper: Tuliao and Velasquez (2017)	359 Filipino university students with 52.09% (*n* = 187) females and 47.91% (*n* = 172) males and mean age of 17.69 (SD 0.97)	Philippines	University-based	Quantitative Cross-sectional study design	Perceived likelihood of seeking help (adapted version) Online counselling attitude scale Self-stigma of seeking help scale Self-concealment scale Interpersonal support evaluation list Inventory of attitudes toward seeking mental health services Loss of face scale Intent to seek counseling inventory General Help Seeking Questionnaire	Moderate*
Vahabi and Wong (2017) [16]	25 Filipino women, age range from 25 to 60 years old	Canada	Community-based	Qualitative Focus group discussion	Semi-structured focus group discussion guide	Good**

*Quality assessment based on the criteria of EPHPP Quality Assessment Tool for quantitative studies

**Quality assessment based on the criteria of CASP Qualitative Checklist for qualitative studies

### Formal help-seeking behaviors

12 studies examined the formal help-seeking behaviors of Filipinos (Table 2), eight of them were from community-based studies and four were from centre-based studies. Nine studies reported on formal help-seeking of overseas Filipinos and three reported on local Filipinos.

**Table 2:** Report on formal help-seeking behaviors

Study	Reports of formal help-seeking behaviors	Outcome measures
Quantitative studies		
Abe-Kim et al. (2007) [10]	2.60% (*n* = 13) used mental health specialty service and 5.20% (*n* = 26) used general medical provider, or total of 7.68% (*n* = 39) sought formal help	Validated research questionnaire from National Latino and Asian American Study
Bernardo and Estrellado (2017)	All participants (*n* = 70) had sought assistance from the women’s shelter	Validated research questionnaire
Cabbigat and Kangas (2017)	39.32% (*n* = 46) sought help within 6 to 12 months following their child’s abuse and/or for child/family problems and 46.15% (*n* = 54) within 18 to 24 months	Validated research questionnaire
David (2010) [34]	No reports on formal help-seeking behavior	
Gong et al. (2003) [13]	4.42% (*n* = 101) used the general medical sector only, 0.61% (*n* = 14) relied solely on mental health care system and 0.35% (*n* = 8) used both mental health specialists and general practitioners, or total of 5.38% (*n* = 123) sought formal help	Validated research questionnaire from Filipino American Community Epidemiological Study
Green and Ayalon (2016) [6]	4.7% reported solely formally, 17.5% (*n* = 15) reported the abuse experience to a social worker, 8.8% (*n* = 7) to a nurse or physician	Validated research questionnaire
Hermannsdottir and Aegisdottir (2016)	12.4% (*n* = 26) participants had prior counseling experience	Validated research questionnaire
Nguyen (2011) [30]	2.2% (*n* = 6) of Filipino participants had sought care from a mental health specialist	Validated research questionnaire from California Health Interview Survey
Tuliao et al. (2016) [12]	22.19% (*n* = 79) sought help from mental health professional	Standardized measures
Qualitative studies		
Hechanova et al. (2013) [31]	10.68% (*n* = 39) sought online counseling services mostly through chat and email with an average of 2 sessions	Semi-structured open-ended questionnaire
Shoultz et al. (2010) [32]	Participants were receiving help from an agency that supports victims of intimate partner violence	Semi-structured interview guide
Straiton et al. (2018) [14]	None of the women had sought help for mental health problems	Semi-structured interview guide with open-ended questions
Vahabi and Wong (2017) [16]	Only one participant indicated that she had used a counselor/ psychotherapist; participants neither used existing mental health services nor knew what services were available to them	Semi-structured focus group discussion guide
Mixed methods		
Ho et al. (2018) [7]	No reports on formal help-seeking behavior	
Thompson et al. (2002) [33]	No reports on formal help-seeking behavior	

*Community-based vs health/social centres* Data from quantitative community studies show that the rates of formal help-seeking behaviors among the Filipino general population ranged from 2.2% [30] to 17.5% [6]. This was supported by reports from qualitative studies where participants did not seek help at all. The frequency of reports of formal help-seeking from studies conducted in crisis centres and online counseling tended to be higher. For instance, the rate of engagement in online counseling among overseas Filipinos was 10.68% [31], those receiving treatment in crisis centers was 39.32% [17] while 100% of participants who were victims of intimate partner violence were already receiving help from a women’s support agency [8, 32].

*Local vs overseas Filipinos’ formal help-seeking* The rate of formal psychological help-seeking of local Filipinos was at 22.19% [12] while overseas rates were lower and ranged from 2.2% of Filipino Americans [30] to 17.5% of Filipinos in Israel [6]. Both local and overseas Filipinos indicated that professional help is sought only as a last resort because they were more inclined to get help from family and friends or lay network [7, 16].

### Attitudes towards formal help-seeking

13 studies reported on participants’ attitudes towards seeking formal help. Seven studies identified family and friends as preferred sources of help [7, 14, 16] rather than mental health specialists and other professionals even when they were already receiving help from them [17, 32]. When Filipinos seek professional help, it is usually done in combination with other sources of care [13] or only used when the mental health problem is severe [14, 16, 33]. Other studies reported that in the absence of social networks, individuals prefer to rely on themselves [32, 33].

*Community-based vs health/social centres* Community-based studies reported that Filipinos have negative attitudes marked by low stigma tolerance towards formal help-seeking [7, 14, 16]. However, different findings were reported by studies conducted in crisis centres. Hechanova et al. found a positive attitude towards help-seeking among users of online counseling [31], whereas Cabbigat and Kangas found that Filipinos in crisis centres still prefer receiving help from religious clergy or family members, with mental health units as the least preferred setting in receiving help [17]. This is supported by the findings of Shoultz and her colleagues who reported that Filipino women did not believe in disclosing their problems to others [32].

*Local vs overseas Filipinos* Filipinos, regardless of location, have negative attitudes towards help-seeking, except later-generation Filipino migrants who have been acculturated in their host countries and tended to have more positive attitudes towards mental health specialists [10, 13, 15, 34]. However, this was only cited in quantitative studies. Qualitative studies reported the general reluctance of both overseas and local Filipinos to seek help.

### Barriers in formal help-seeking

All 15 studies examined a range of barriers in psychological help-seeking (Table 3). The most commonly endorsed barriers were: (1) financial constraints due to high cost of service, lack of health insurance, or precarious employment condition; (2) self-stigma, with associated fear of negative judgment, sense of shame, embarrassment and being a disgrace, fear of being labeled as ‘crazy’, self-blame and concern for loss of face; and (3) social stigma that puts the family’s reputation at stake or places one’s cultural group in bad light.

**Table 3:** Key themes on barriers to formal help-seeking

Key barrier themes	Studies on local Filipinos (*n* = 4)	Studies on overseas Filipinos (*n* = 11)	Total (*n* = 15)
(A) Systemic, structural and economic barriers			
1. Financial constraints (e.g., high cost of service, lack of health insurance, fear of losing job, precarious nature of employment)	2	10	12 (80%)
2. Inaccessibility of mental health services (e.g., lack of familiarity or information on available mental health services, different structure of mental health system, lack of time, geographical dispersal)	2	6	8 (53%)
3. Immigration/Residency status (e.g., nativity, fear of deportation)	N/A	7	7 (47%)
(B) Socio-cultural barriers			
1. Social stigma (e.g., attack on family reputation or negative perception of one’s cultural group, preservation of the family’s dignity, fear of social exclusion, being labelled as ‘crazy’)	2	8	10 (67%)
2. Sense of religiosity (e.g., preference for religious clergy, strong religious belief, reliance on faith organizations) and/or spirituality	2	6	8 (53%)
3. Language difficulty (e.g., lack of language proficiency in the host country)	0	6	6 (40%)
4. Adherence to Asian cultural values of conformity; lack of acculturation	1	4	5 (33%)
5. Use of alternative health care (e.g., indigenous healing methods, use of herbal medicines, consultation with elders in the community)	0	2	2 (13%)
(C) Psychosocial barriers			
1. Self-stigma (e.g., concern for loss of face, sense of shame or embarrassment, fear of being judged negatively, fear of negative reactions from family or friends, sense of being a disgrace, self-blame, fear of being labeled as ‘crazy’, sense of being weak)	2	9	11 (73%)
2. Influence of social support/network (e.g., presence of and preference for family and friends as source of help, lack of friends to provide influence)	3	6	9 (60%)
3. Previous negative experience of help-seeking (e.g., experience of discrimination, lack of trust on or rapport with healthcare provider)	1	7	8 (53%)
4. Concerns on confidentiality and privacy, treatment fears e.g., concerns on trustworthiness or competence of the mental health care provider, effect of medication)	2	5	7 (47%)
5. Lack of awareness of mental health need (e.g., low perception of distress; normalization of mental health problems)	1	6	7 (47%)
6. Misconceptions about mental illness (i.e., on nature, causes and effects of mental health problems)	2	4	6 (40%)
7. Sense of self-reliance (e.g., perceived resilience, coping ability, sense of self-responsibility)	0	3	3 (20%)
8. Fear of hurting or becoming burden to others	0	3	3 (20%)

*Local vs overseas Filipinos* In studies conducted among overseas Filipinos, strong adherence to Asian values of conformity to norms is an impediment to help-seeking but cited only in quantitative studies [10, 13, 15, 34] while perceived resilience, coping ability or self-reliance was mentioned only in qualitative studies [14, 16, 33]. Other common barriers to help-seeking cited by overseas Filipinos were inaccessibility of mental health services, immigration status, sense of religiosity, language problem, experience of discrimination and lack of awareness of mental health needs [10, 13, 18, 34]. Self-reliance and fear of being a burden to others as barriers were only found among overseas Filipinos [6, 16, 32]. On the other hand, local Filipinos have consistently cited the influence of social support as a hindrance to help-seeking [7, 17].

Stigmatized attitude towards mental health and illness was reported as topmost barriers to help-seeking among overseas and local Filipinos. This included notions of mental illness as a sign of personal weakness or failure of character resulting to loss of face. There is a general consensus in these studies that the reluctance of Filipinos to seek professional help is mainly due to their fear of being labeled or judged negatively, or even their fear of fueling negative perceptions of the Filipino community. Other overseas Filipinos were afraid that having mental illness would affect their jobs and immigration status, especially for those who are in precarious employment conditions [6, 16].

### Facilitators of formal help-seeking

All 15 studies discussed facilitators of formal help-seeking, but the identified enablers were few (Table 4). Among the top and commonly cited factors that promote help-seeking are: (1) perceived severity of the mental health problem or awareness of mental health needs; (2) influence of social support, such as the presence/absence of family and friends, witnessing friends seeking help, having supportive friends and family who encourage help-seeking, or having others taking the initiative to help; and (3) financial capacity.

**Table 4:** Key themes on facilitators in help-seeking

Key facilitator themes	Studies on local Filipinos (*n* = 4)	Studies on overseas Filipinos (*n* = 11)	Total (*n* = 15)
(A) Systemic and economic facilitators			
1. Financial capacity (e.g., higher socio-economic status, employment status, medical insurance, higher income)	1	4	5 (33%)
2. Immigration/Residency status (e.g., nativity status, being US-born Filipino American, later-generation immigrants)	N/A	3	3 (20%)
3. Accessibility of mental health services (e.g., technological access, presence of technical infrastructure, perception of ease of use, familiarity with health care services)	0	2	2 (13%)
(B) Socio-cultural facilitators			
1. Language proficiency (e.g., bilingualism, proficiency of the language of host culture)	0	4	4 (27%)
2. Lower adherence to Asian cultural values (e.g., higher levels of acculturation/assimilation)	0	3	3 (20%)
3. Higher level of spirituality	0	2	2 (13%)
(C) Psycho-social facilitators			
1. Perception of distress (e.g., awareness of mental health need; higher severity of mental health problems)	2	5	7 (47%)
2. Influence of social support (presence/absence of family and friends, witnessing friends seeking help, having supportive friends and family who encourage help-seeking, others taking the initiative to help)	2	4	6 (40%)
3. Self-stigma tolerance (e.g., concern for loss of face, lower/higher tolerance of stigma)	1	3	4 (27%)
4. Previous experience in help-seeking (e.g., positive experience with mental health professionals, establishing rapport with mental health providers)	2	2	4 (27%)
5. Higher awareness of mental health issues	2	0	2 (13%)
6. Sense of anonymity	1	1	2 (13%)

*Local vs overseas Filipinos* Studies on overseas Filipinos frequently cited financial capacity, immigration status, language proficiency, lower adherence to Asian values and stigma tolerance as enablers of help-seeking [15, 30, 32, 34], while studies done on local Filipinos reported that awareness of mental health issues and previous positive experience of seeking help serve as facilitator [7, 12].

*Community-based vs health/social centres* Those who were receiving help from crisis centres mentioned that previous positive experience with mental health professionals encouraged their formal help-seeking [8, 17, 31]. On the other hand, community-based studies cited the positive influence of encouraging family and friends as well as higher awareness of mental health problems as enablers of help-seeking [12, 14, 16].

## Discussion

To the best of our knowledge, this is the first systematic review conducted on psychological help-seeking among Filipinos, including its barriers and facilitators. The heterogeneity of participants (e.g., age, gender, socio-economic status, geographic location or residence, range of mental health problems) was large.

*Filipino mental health help-seeking behavior and attitudes* The rate of mental health problems appears to be high among Filipinos both local and overseas, but the rate of help-seeking is low. This is consistent with findings of a study among Chinese immigrants in Australia which reported higher psychological distress but with low utilization of mental health services [35]. The actual help-seeking behavior of both local and overseas Filipinos recorded at 10.72% (*n* = 461) is lower than the 19% of the general population in the US [36] and 16% in the United Kingdom (UK) [37], and even far below the global prevalence rate of 30% of people with mental illness receiving treatment [38]. This finding is also comparable with the low prevalence rate of mental health service use among the Chinese population in Hong Kong [39] and in Australia [35], Vietnamese immigrants in Canada [30], East Asian migrants in North America [41] and other ethnic minorities [42] but is in sharp contrast with the increased use of professional help among West African migrants in The Netherlands [43].

Most of the studies identified informal help through family and friends as the most widely utilized source of support, while professional service providers were only used as a last resort. Filipinos who are already accessing specialist services in crisis centres also used informal help to supplement professional help. This is consistent with reports on the frequent use of informal help in conjunction with formal help-seeking among the adult population in UK [44]. However, this pattern contrasts with informal help-seeking among African Americans who are less likely to seek help from social networks of family and friends [45]. Filipinos also tend to use their social networks of friends and family members as ‘go-between’ [46] for formal help, serving to intercede between mental health specialists and the individual. This was reiterated in a study by Shoultz et al. (2009) in which women who were victims of violence are reluctant to report the abuse to authorities but felt relieved if neighbours and friends would interfere for professional help in their behalf [32].

Different patterns of help-seeking among local and overseas Filipinos were evident and may be attributed to the differences in the health care system of the Philippines and their host countries. For instance, the greater use of general medical services by overseas Filipinos is due to the gatekeeper role of general practitioners (GP) in their host countries [47] where patients have to go through their GPs before they get access to mental health specialists. In contrast, local Filipinos have direct access to psychiatrists or psychologists without a GP referral. Additionally, those studies conducted in the Philippines were done in urban centers where participants have greater access to mental health specialists. While Filipinos generally are reluctant to seek help, later-generation overseas Filipinos have more positive attitudes towards psychological help-seeking. Their exposure and acculturation to cultures that are more tolerant of mental health stigma probably influenced their more favorable attitude [41, 48].

*Prominent barrier themes in help-seeking* Findings of studies on frequently endorsed barriers in psychological help-seeking are consistent with commonly reported impediments to health care utilization among Filipino migrants in Australia [49] and Asian migrants in the US [47, 50]. The same barriers in this review, such as preference for self-reliance as alternative coping strategy, poor mental health awareness, perceived stigma, are also identified in mental health help-seeking among adolescents and young adults [51] and among those suffering from depression [52].

Social and self-stigmatizing attitudes to mental illness are prominent barriers to help-seeking among Filipinos. Social stigma is evident in their fears of negative perception of the Filipino community, ruining the family reputation, or fear of social exclusion, discrimination and disapproval. Self-stigma manifests in their concern for loss of face, sense of shame or embarrassment, self-blame, sense of being a disgrace or being judged negatively and the notion that mental illness is a sign of personal weakness or failure of character [16]. The deterrent role of mental health stigma is consistent with the findings of other studies [51, 52]. Overseas Filipinos who are not fully acculturated to the more stigma-tolerant culture of their host countries still hold these stigmatizing beliefs. There is also a general apprehension of becoming a burden to others.

Practical barriers to the use of mental health services like accessibility and financial constraints are also consistently rated as important barriers by Filipinos, similar to Chinese Americans [53]. In the Philippines where mental health services are costly and inaccessible [54], financial constraints serve as a hindrance to formal help-seeking, as mentioned by a participant in the study of Straiton and his colleagues, “In the Philippines… it takes really long time to decide for us that this condition is serious. We don’t want to use our money right away” [14, p.6]. Local Filipinos are confronted with problems of lack of mental health facilities, services and professionals due to meager government spending on health. Despite the recent ratification of the Philippines’ Mental Health Act of 2018 and the Universal Health Care Act of 2019, the current coverage for mental health services provided by the Philippine Health Insurance Corporation only amounts to US$154 per hospitalization and only for acute episodes of mental disorders [55]. Specialist services for mental health in the Philippines are restricted in tertiary hospitals located in urban areas, with only one major mental hospital and 84 psychiatric units in general hospitals [1].

Overseas Filipinos cited the lack of health insurance and immigration status without health care privileges as financial barrier. In countries where people have access to universal health care, being employed is a barrier to psychological help-seeking because individuals prefer to work instead of attending medical check-ups or consultations [13]. Higher income is also associated with better mental health [56] and hence, the need for mental health services is low, whereas poor socio-economic status is related to greater risk of developing mental health problems [57, 58]. Lack of familiarity with healthcare system in host countries among new Filipino migrants also discourages them from seeking help.

Studies have shown that reliance on, and accessibility of sympathetic, reliable and trusted family and friends are detrimental to formal help-seeking since professional help is sought only in the absence of this social support [6, 8]. This is consistent with the predominating cultural values that govern Filipino interpersonal relationships called kapwa (or shared identity) in which trusted family and friends are considered as “hindi-ibang-tao” (one-of-us/insider), while doctors or professionals are seen as “ibang-tao” (outsider) [59]. Filipinos are apt to disclose and be more open and honest about their mental illness to those whom they considered as “hindi-ibang-tao” (insider) as against those who are “ibang-tao” (outsider), hence their preference for family members and close friends as source of informal help [59]. For Filipinos, it is difficult to trust a mental health specialist who is not part of the family [60].

Qualitative studies in this review frequently mentioned resilience and self-reliance among overseas Filipinos as barriers to help-seeking. As an adaptive coping strategy for adversity [61], overseas Filipinos believe that they were better equipped in overcoming emotional challenges of immigration [16] without professional assistance [14]. It supports the findings of studies on overseas Filipino domestic workers who attributed their sense of well-being despite stress to their sense of resilience which prevents them from developing mental health problems [62] and among Filipino disaster survivors who used their capacity to adapt as protective mechanism from experience of trauma [63]. However, self-reliant individuals also tend to hold stigmatizing beliefs on mental health and as such resort to handling problems on their own instead of seeking help [51, 64].

*Prominent facilitator themes in help-seeking* In terms of enablers of psychological help-seeking, only a few facilitators were mentioned in the studies, which supported findings in other studies asserting that factors that promote help-seeking are less often emphasized [42, 51].

Consistent with other studies [44, 49], problem severity is predictive of intention to seek help from mental health providers [18, 30] because Filipinos perceive that professional services are only warranted when symptoms have disabling effects [5, 53]. As such, those who are experiencing heightened emotional distress were found to be receptive to intervention [17]. In most cases, symptom severity is determined only when somatic or behavioral symptoms manifest [13] or occupational dysfunction occurs late in the course of the mental illness [65]. This is most likely due to the initial denial of the problem [66] or attempts at maintaining normalcy of the situation as an important coping mechanism [67]. Furthermore, this poses as a hindrance to any attempts at early intervention because Filipinos are likely to seek professional help only when the problem is severe or has somatic manifestations. It also indicates the lack of preventive measure to avert any deterioration in mental health and well-being.

More positive attitudes towards help-seeking and higher rates of mental health care utilization have been found among later-generation Filipino immigrants or those who have acquired residency status in their host country [10, 15]. Immigration status and length of stay in the host country are also associated with language proficiency, higher acculturation and familiarity with the host culture that are more open to discussing mental health issues [13], which present fewer barriers in help-seeking. This is consistent with facilitators of formal help-seeking among other ethnic minorities, such as acculturation, social integration and positive attitude towards mental health [43].

*Cultural context of Filipinos’ reluctance to seek help* Several explanations have been proposed to account for the general reluctance of Filipinos to seek psychological help. In Filipino culture, mental illness is attributed to superstitious or supernatural causes, such as God’s will, witchcraft, and sorcery [68, 69], which contradict the biopsychosocial model used by mental health care professionals. Within this cultural context, Filipinos prefer to seek help from traditional folk healers who are using religious rituals in their healing process instead of availing the services of professionals [70, 71]. This was reaffirmed by participants in the study of Thompson and her colleagues who said that “psychiatrists are not a way to deal with emotional problems” [74, p.685]. The common misconception on the cause and nature of mental illness, seeing it as temporary due to cold weather [14] or as a failure in character and as an individual responsibility to overcome [16, 72] also discourages Filipinos from seeking help.

Synthesis of the studies included in the review also found conflicting findings on various cultural and psychosocial influences that served both as enablers and deterrents to Filipino help-seeking, namely: (1) level of spirituality; (2) concern on loss of face or sense of shame; and (3) presence of social support.

*Level of spirituality* Higher spirituality or greater religious beliefs have disparate roles in Filipino psychological help-seeking. Some studies [8, 14, 16] consider it a hindrance to formal help-seeking, whereas others [10, 15] asserted that it can facilitate the utilization of mental health services [15, 73]. Being predominantly Catholics, Filipinos had drawn strength from their religious faith to endure difficult situations and challenges, accordingly ‘leaving everything to God’ [74] which explains their preference for clergy as sources of help instead of professional mental health providers. This is connected with the Filipino attribution of mental illness to spiritual or religious causes [62] mentioned earlier. On the contrary, Hermansdottir and Aegisdottir argued that there is a positive link between spirituality and help-seeking, and cited connectedness with host culture as mediating factor [15]. Alternately, because higher spirituality and religiosity are predictors of greater sense of well-being [75], there is, thus, a decreased need for mental health services.

*Concern on loss of face or sense of shame* The enabler/deterrent role of higher concern on loss of face and sense of shame on psychological help-seeking was also identified. The majority of studies in this review asserted the deterrent role of loss of face and stigma consistent with the findings of other studies [51], although Clement et al. stated that stigma is the fourth barrier in deterring help-seeking [76]. Mental illness is highly stigmatized in the Philippines and to avoid the derogatory label of ‘crazy’, Filipinos tend to conceal their mental illness and consequently avoid seeking professional help. This is aligned with the Filipino value of *hiya* (sense of propriety) which considers any deviation from socially acceptable behavior as a source of shame [11]. The stigmatized belief is reinforced by the notion that formal help-seeking is not the way to deal with emotional problems, as reflected in the response of a Filipino participant in the study by Straiton et. al., “It has not occurred to me to see a doctor for that kind of feeling” [14, p.6]. However, other studies in this review [12, 13] posited contrary views that lower stigma tolerance and higher concern for loss of face could also motivate psychological help-seeking for individuals who want to avoid embarrassing their family. As such, stigma tolerance and loss of face may have a more nuanced influence on help-seeking depending on whether the individual avoids the stigma by not seeking help or prevent the stigma by actively seeking help.

*Presence of social support* The contradictory role of social networks either as helpful or unhelpful in formal help-seeking was also noted in this review. The presence of friends and family can discourage Filipinos from seeking professional help because their social support serves as protective factor that buffer one’s experience of distress [77, 78]. Consequently, individuals are less likely to use professional services [42, 79]. On the contrary, other studies have found that the presence of friends and family who have positive attitudes towards formal help-seeking can promote the utilization of mental health services [8, 80]. Friends who sought formal help and, thus, serve as role models [14], and those who take the initiative in seeking help for the distressed individual [32] also encourage such behavior. Thus, the positive influence of friends and family on mental health and formal help-seeking of Filipinos is not merely to serve only as emotional buffer for stress, but to also favourably influence the decision of the individual to seek formal help.

## Research implications of findings

This review highlights particular evidence gaps that need further research: (1) operationalization of help-seeking behavior as a construct separating intention and attitude; (2) studies on actual help-seeking behavior among local and overseas Filipinos with identified mental health problems; (3) longitudinal study on intervention effectiveness and best practices; (4) studies that triangulate findings of qualitative studies with quantitative studies on the role of resilience and self-reliance in help-seeking; and (5) factors that promote help-seeking.

Some studies in this review reported help-seeking intention or attitude as actual behaviors even though they are separate constructs, hence leading to reporting biases and misinterpretations. For instance, the conflicting findings of Tuliao et al. [12] on the negative association of loss of face with help-seeking attitude and the positive association between loss of face and intention to seek help demonstrate that attitudes and intentions are separate constructs and, thus, need further operationalization. Future research should strive to operationalize concretely these terms through the use of robust measurement tools and systematic reporting of results. There is also a lack of data on the actual help-seeking behaviors among Filipinos with mental illness as most of the reports were from the general population and on their help-seeking attitudes and intentions. Thus, research should focus on those with mental health problems and their actual utilization of healthcare services to gain a better understanding of how specific factors prevent or promote formal help-seeking behaviors.

Moreover, the majority of the studies in this review were descriptive cross-sectional studies, with only one cohort analytic study. Future research should consider a longitudinal study design to ensure a more rigorous and conclusive findings especially on testing the effectiveness of interventions and documenting best practices. Because of the lack of quantitative research that could triangulate the findings of several qualitative studies on the detrimental role of resilience and self-reliance, quantitative studies using pathway analysis may help identify how these barriers affect help-seeking. A preponderance of studies also focused on discussing the roles of barriers in help-seeking, but less is known about the facilitators of help-seeking. For this reason, factors that promote help-seeking should be systematically investigated.

## Practice, service delivery and policy implications

Findings of this review also indicate several implications for practice, service delivery, intervention and policy. Cultural nuances that underlie help-seeking behavior of Filipinos, such as the relational orientation of their interactions [81], should inform the design of culturally appropriate interventions for mental health and well-being and improving access and utilization of health services. Interventions aimed at improving psychological help-seeking should also target friends and family as potential and significant influencers in changing help-seeking attitude and behavior. They may be encouraged to help the individual to seek help from the mental health professional. Other approaches include psychoeducation that promotes mental health literacy and reduces stigma which could be undertaken both as preventive and treatment strategies because of their positive influence on help-seeking. Strategies to reduce self-reliance may also be helpful in encouraging help-seeking.

This review also has implications for structural changes to overcome economic and other practical barriers in Filipino seeking help for mental health problems. Newly enacted laws on mental health and universal healthcare in the Philippines may jumpstart significant policy changes, including increased expenditure for mental health treatment.

Since lack of awareness of available services was also identified as significant barrier, overseas Filipinos could be given competency training in utilizing the health care system of host countries, possibly together with other migrants and ethnic minorities. Philippine consular agencies in foreign countries should not merely only resort to repatriation acts, but could also take an active role in service delivery especially for overseas Filipinos who experience trauma and/or may have immigration-related constraints that hamper their access to specialist care.

## Limitations of findings

A crucial limitation of studies in this review is the use of different standardized measures of help-seeking that render incomparable results. These measures were western-based inventories, and only three studies mentioned using cultural validation, such as forward-and-back-translations, to adapt them to cross-cultural research on Filipino participants. This may pose as a limitation on the cultural appropriateness and applicability of foreign-made tests [73] in capturing the true essence of Filipino experience and perspectives [74]. Additionally, the majority of the studies used non-probability sampling that limits the generalizability of results. They also failed to measure the type of assistance or actual support sought by Filipinos, such as psychoeducation, referral services, supportive counseling or psychotherapy, and whether or not they are effective in addressing mental health concerns of Filipinos. Another inherent limitation of this review is the lack of access to grey literature, such as thesis and dissertations published in other countries, or those published in the Philippines and are not available online. A number of studies on multi-ethnic studies with Filipino participants do not provide disaggregated data, which limits the scope and inclusion of studies in this review.

## Conclusion

This review has confirmed the low utilization of mental health services among Filipinos regardless of their locations, with mental health stigma as a primary barrier resilience and self-reliance as coping strategies were also cited, especially in qualitative studies, but may be important in addressing issues of non-utilization of mental health services. Social support and problem severity were cited as prominent facilitators in help-seeking. However, different structural, cultural and practical barriers and facilitators of psychological help-seeking between overseas and local Filipinos were also found.
